# Magnetic Yeast Glucan Particles for Antibody-Free Separation of Viable Macrophages from *Drosophila melanogaster*

**DOI:** 10.1021/acsbiomaterials.3c01199

**Published:** 2023-12-04

**Authors:** Gabriela Krejčová, Ivan Saloň, Vojtěch Klimša, Pavel Ulbrich, Ayse Beyza Aysan, Adam Bajgar, František Štěpánek

**Affiliations:** † Department of Molecular Biology and Genetics, Faculty of Sciences, University of South Bohemia, Branišovská 1160/31, 37005 České Budějovice, Czech Republic; ‡ Department of Chemical Engineering, University of Chemistry and Technology Prague, Technická 5, 166 28 Prague 6, Czech Republic; § Department of Biochemistry and Microbiology, University of Chemistry and Technology, Prague, Technická 5, 166 28 Prague 6, Czech Republic

**Keywords:** β-glucan particles, iron oxide nanoparticles, spray drying, cell separation, phagocytosis

## Abstract

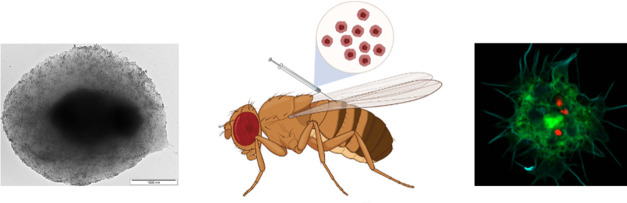

Currently available methods for cell separation are generally based on fluorescent labeling using either endogenously expressed fluorescent markers or the binding of antibodies or antibody mimetics to surface antigenic epitopes. However, such modification of the target cells represents potential contamination by non-native proteins, which may affect further cell response and be outright undesirable in applications, such as cell expansion for diagnostic or therapeutic applications, including immunotherapy. We present a label- and antibody-free method for separating macrophages from living *Drosophila* based on their ability to preferentially phagocytose whole yeast glucan particles (GPs). Using a novel deswelling entrapment approach based on spray drying, we have successfully fabricated yeast glucan particles with the previously unachievable content of magnetic iron oxide nanoparticles while retaining their surface features responsible for phagocytosis. We demonstrate that magnetic yeast glucan particles enable macrophage separation at comparable yields to fluorescence-activated cell sorting without compromising their viability or affecting their normal function and gene expression. The use of magnetic yeast glucan particles is broadly applicable to situations where viable macrophages separated from living organisms are subsequently used for analyses, such as gene expression, metabolomics, proteomics, single-cell transcriptomics, or enzymatic activity analysis.

## Introduction

Cell manipulation and processing are crucial operations in biomedical research when working with living animals, tissues, and cells. Doing it in a lean and effective manner without compromising cellular functions is key to the further use of separated cells. Currently used techniques include micro pipetting,^1^ microfluidics,^2^ high-gradient magnetic cell sorting,^3^ and predominantly fluorescence-activated cell sorting (FACS).^4^ Existing methods are generally based on fluorescent labeling of the target cells using either endogenously expressed fluorescent markers or the binding of antibodies or antibody mimetics to surface antigenic epitopes. In the case of magnetic cell sorting, the currently used methods use antigen-coupled magnetic nanoparticles that bind to the cell surface. While these approaches are perfectly acceptable in many applications such as ex post metabolomic analysis, there are also situations where the addition of non-native proteins to the separated cells is undesirable,^5,6^ particularly if the cells are to be used for immuno-analysis and diagnostic or therapeutic purposes.^7^ The cell viability can be compromised, and normal cellular functions including immune response can be affected by phenomena such as antigen shedding.^8^ From the regulatory perspective in cell therapy, the contamination of the therapeutic product by nonautologous or adventitious proteins can be problematic.

Whole yeast glucan particles (GPs) are porous polysaccharide shells predominantly formed by β-glucans, obtained from common baker’s yeast by a series of washing and extraction steps.^9^ Although most cellular components of the original yeast are removed, GPs retain surface structural features that make them readily recognized by dectin-1 receptors of immune cells and actively phagocytosed. This property of GPs has been well documented both ex vivo^10,11^ and in vivo.^12,13^ Owing to their immunogenicity and porous nature, GPs lend themselves as vehicles for the encapsulation and targeted delivery of various bioactive substances.^14−17^ Proposed diagnostic and therapeutic applications of GPs include their use as vaccine adjuvants,^18^ as immuno-active drug delivery systems for the treatment of inflammatory bowel disease, as a means of improving the bioavailability of poorly soluble drugs via lymphatic transport,^13^ or as contrast agents for imaging.^19,20^ It has been recently shown that GPs injected into living *Drosophila* are rapidly distributed through the hemolymph and selectively taken up by macrophages without compromising their normal function.^9^

This feature of GPs could be used for label-free macrophage separation by a magnetic field, but achieving sufficiently high magnetic response of GPs without compromising their morphology and surface molecular motifs has so far eluded the scientific community. In the present work, we introduce a novel method that yields composite GPs with an unprecedentedly strong response to magnetic field while retaining their structural and functional properties. The method is based on encapsulating independently prepared magnetic iron oxide nanoparticles (IONs) into GPs by spray drying. A solvent temporarily swells the polysaccharide GP shell, enabling colloidally stable magnetic IONs to diffuse into the inner structure. By rapid solvent evaporation during spray drying, the polysaccharide shell deswells and magnetic nanoparticles are irreversibly trapped within the GPs at a high concentration, while a native GP surface is preserved. We report a comprehensive physicochemical characterization of magnetic GPs and demonstrate their in vivo biodistribution, cell uptake, and successful application for magnetic separation. Furthermore, we show that the normal function and gene expression profiles of the separated macrophages are preserved.

## Materials and Methods

### Preparation of Yeast Glucan Particles

GPs were obtained from baker’s yeast (*Saccharomyces cerevisiae*) using a series of washing and extraction steps as reported previously.^14^ 25 g portion of baker’s yeast was added into 100 mL of 1 M NaOH and mixed to form a suspension, and the material was heated for 1 h at 90 °C and then centrifuged at 14,500 g for 5 min (Dynamica Velocity 14, Austria). The supernatant was discarded, and this step was repeated twice. The processed alkali-insoluble solids were then mixed with 10 mL of HCl solution (pH 4.5), heated to 75 °C for 2 h, and then centrifuged at 14,500 g for 5 min. The insoluble solids were washed 3 times in deionized water, 4 times in isopropanol, and finally 2 times in acetone. Each washing step was followed by centrifugation at 14,500 g for 5 min. The final product was freeze-dried to form a white dry powder and stored in a refrigerator for further use.

### Preparation of Yeast GPs Modified with Rhodamine B

As a reference for visualization experiments, Rhodamine B-modified yeast GPs (GP-RhodB) were prepared by dispersing 50 mg of glucan particles in 10 mL of 0.1 M carbonate-bicarbonate buffer with pH 9.2 containing 1 mg of Rhodamine B isothiocyanate dissolved in 500 μL of ethanol in a round-bottom flask. The suspension was sonicated in a sonication bath for 15 min. The suspension was then kept at 37 °C for 12 h under constant magnetic stirring at 500 rpm. The content of the reaction mixture was then washed 16 times and centrifuged for 3 min at 6000 g. The supernatant-containing unreacted material was discarded, and the obtained pellet was freeze-dried and stored in a refrigerator for further use.

### Synthesis of Dextran-Coated Iron Oxide Nanoparticles

Dextran-coated IONs were synthesized as follows: 0.75 g of iron(III) chloride hexahydrate (Sigma-Aldrich) and 0.375 g of iron(II) chloride tetrahydrate (Sigma-Aldrich) were dissolved in 15 mL of deionized water and kept in a 100 mL three-neck flask equipped with a reverse cooler in a nitrogen atmosphere under vigorous stirring. 500 mg of 70 kDa dextran (Sigma-Aldrich) dissolved in 25 mL of deionized water was added, the mixture was then heated to 85 °C, and 2.5 mL of 25% NH_4_OH (Penta) was added dropwise into the reaction vessel. The reaction mixture was kept at 85 °C for 1 h and then cooled to room temperature. The nanoparticles were separated by magnetic decantation and washed 3 times with deionized water. The nanoparticle suspension was subsequently dialyzed for 24 h against deionized water. The dialysate was sonicated for 10 min in a sonication bath and centrifuged at 1500 g for 5 min to remove any larger agglomerates. After centrifugation, the supernatant was filtered by a 0.2 μm PVDF (polyvinylidene difluoride) filter to obtain a nanoparticle suspension.^21,22^

### Preparation of Magnetic Yeast Glucan Particles

**Figure 1:**
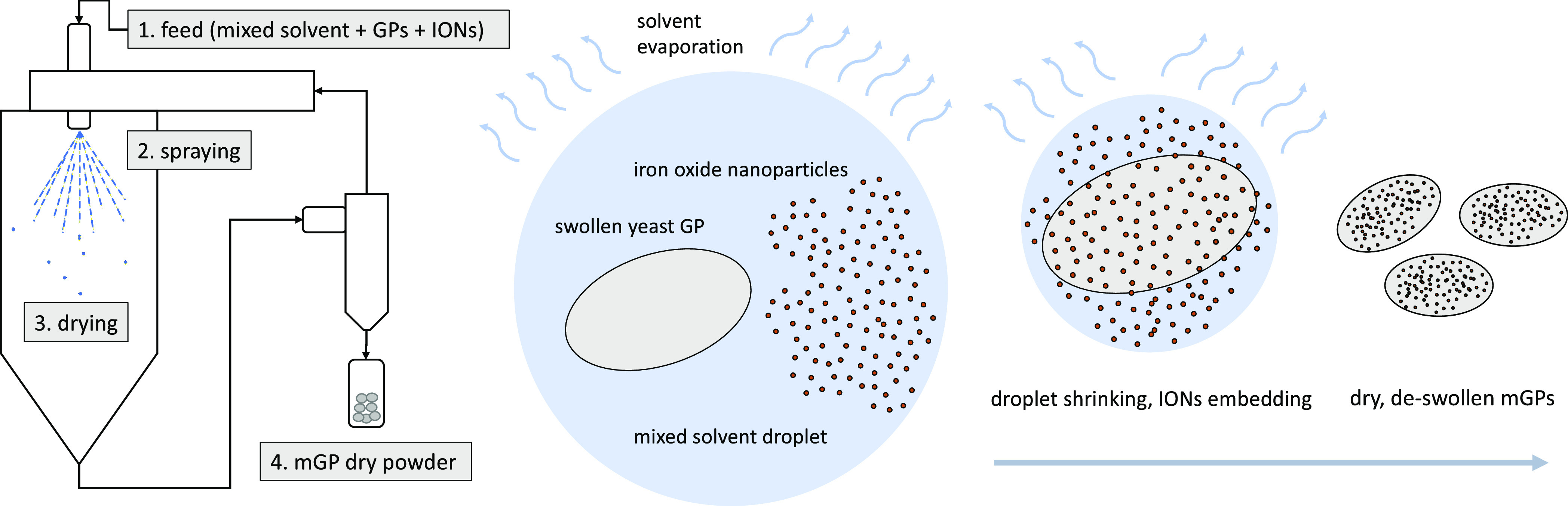
Scheme of the mGP preparation process by spray drying. Left: overall process scheme. Right: mechanism of IONs embedding into GPs during droplet evaporation in the spray drying chamber.

Composite magnetic yeast GPs (mGPs) containing dextran-coated IONs were prepared by spray drying, as shown schematically in Figure 1. 100 mg of yeast GPs (either plain GPs or GP-RhodB) was dispersed and homogenized by ULTRA-TURRAX in a prepared mixture containing 500 μL of IONs (0.215 mg/mL), 25 mL of deionized water, and 75 mL of 96% ethanol. After dispersing, the suspension was immediately spray-dried using a Mini Spray Dryer B-290 (Büchi, Switzerland) operated in an inert loop under a N_2_ atmosphere. Spray drying was conducted using a 1.4 mm diameter, a 2-fluid nozzle, and operating conditions consisting of 120 °C inlet temperature, 5 mL/min suspension feed rate, and 800 L/h (50%) N_2_ flow rate.^15^ The outlet temperature was 70–75 °C.

### Particle Size Analysis

The size distribution of the prepared magnetic nanoparticles (IONs) was evaluated by dynamic light scattering (DLS), using a Zetasizer Nano-ZS (Malvern Instruments, UK). Before the measurement, 10 μL of the sample was added to 2 mL of deionized water, filtered by a 0.2 μm PVD filter, and placed into a disposable cuvette. The size distribution of GPs, mGPs, and mGPs-RhodB was evaluated by the static light scattering method using the Horiba Partica LA 950/S2 instrument. Prior to the measurement, the particle suspension was sonicated by Sonopuls HD 3100 (Bandelin Electronic) for 5 min at 25 W without pulses.

### Electron Microscopy

The surface morphology and shape of GPs and mGPs were examined by a scanning electron microscope Jeol JCM- 5700. Samples were sputter-coated (Emitech K550X) with a 5 nm layer of gold prior to scanning electron microscopy (SEM) analysis. Transmission electron microscopy (TEM) Jeol JEM-1010 was used for the examination of the size and surface morphology of IONs and mGPs, without any staining procedure prior to the analyses. The elemental analysis of mGPs was determined by energy-dispersive X-ray spectroscopy (EDX) using the Thermo Scientific Phenom ProX desktop SEM with Phenom EDS software and semiautomated scanning option.

### Atomic Absorption Spectroscopy

The iron content in mGP samples and in solution was evaluated by atomic absorption spectroscopy (AAS) using Agilent 280FS AA with a flame atomization technique. The Fe (Flame) method at 248.3 nm was used with a flame type: acetylene–air.

### X-ray Powder Diffraction

Tthe crystallinity and the presence of iron oxide in composite mGPs were evaluated by recording the diffraction intensities of the samples from 6° to 110° 2θ angle using a PANaytical X’Pert PRO with a High Score Plus diffractometer. Data evaluation was performed in the software package HighScore Plus 4.0.

### *Drosophila melanogaster* Strains and Culture

The flies were raised on a standard diet containing cornmeal (80 g/L), sucrose (50 g/L), yeast (40 g/L), agar (10.433 g/L), and 10% methylparaben (16.7 mL/L) and were maintained in a humidity-controlled environment with a natural 12 h/12 h light/dark cycle at 25 °C. We used CrqGal4 > GFP fly line for the visualization of macrophages. This strain carries a macrophage-specific driver Crq Gal4 and reporter gene (enhanced green fluorescent protein eGFP) under the control of artificial UAS promoter (genotype *w*^*1118*^*/ w*^*1118*^*; Crq-Gal4, UAS-2xeGFP/Crq-Gal4, UAS-2xeGFP*).

### Injection of Flies

The suspension of IONs, mGPs, or mGPs-RhodB was prepared by sonication on an ice bath for 5 min at 25 W and vortexed just before injection to ensure well-dispersed particles. CrqGal4 > GFP male flies were anaesthetized using CO_2_ and injected with 50 nL of 0.1% (w/w) suspension, in case of mGPs or mGPs-RhodB, into the ventrolateral side of the abdomen using an Eppendorf Femtojet microinjector.

### Visualization of Magnetic Yeast GPs' Distribution In Vivo

To analyze magnetic particle distribution in *Drosophila*, CrqGal4 > GFP flies were injected with 50 nL of 0.1% (w/w) mGPs or mGPs-RhodB. After 45 min, the fly abdomens were opened in 4% PFA (Polysciences) in PBS and fixed for 20 min. Subsequently, the tissues were washed in PBS. Aqua Polymount (Polysciences) was used to mount the sample. The samples were imaged using an inverted fluorescent microscope (Olympus IX71) or a confocal microscope (Olympus FluoView 1000).

### Visualization of mGPs Uptake by *Drosophila* Phagocytes

To visualize mGPs' uptake by *Drosophila*-phagocytosing cells, we prepared samples for confocal and both SEM and TEM. For the analysis using a confocal microscope, CrqGal4 > GFP flies were injected with 50 nL of 0.1% (w/w) mGPs-RhodB. After 45 min, the fly abdomens were opened in a drop of PBS on an imaging slide in order to wash up the macrophages, which were let to attach to the imaging slide for 25 min. Subsequently, the macrophages were fixed with 4% PFA (Polysciences) in PBS. After 20 mi, the samples were stained with Alexa Fluor Plus 405 Phalloidin (Invitrogen) for 40 min. Aqua Polymount (Polysciences) was used to mount the sample. Macrophages were imaged using an Olympus FluoView 3000 confocal microscope.

For the SEM analysis, CrqGal4 > GFP flies were injected with 50 nL of 0.1% (w/w) mGPs. After 45 min, the fly abdomens were opened in PBS and fixed in 2.5% glutaraldehyde in 0.1 M phosphate buffer (pH = 7.2) for 1 week at 4 °C. Subsequently, the opened abdomens were dehydrated through an acetone series and dried to critical point by point dryer CPD 2 (Pelco TM) and attached to an aluminum target. For contrasting, the samples were coated with gold by using a sputter-coated E5100 (Polar Equipment Ltd.). Macrophages were examined with JEOL SEM JSM 7401F. Electron images were false colorized in Adobe Photoshop software.

For the TEM analysis, CrqGal4 > GFP flies were injected with 50 nL of 0.1% (w/w) mGPs. After 45 min, the fly abdomens were cut off and placed in 2.5% glutaraldehyde in 0.1 M phosphate buffer (pH = 7.2) for 1 week at 4 °C. Subsequently, the samples for TEM were postfixed in osmium tetroxide for 2 h at 4 °C, washed at 4 °C, dehydrated through an acetone serie, and embedded in EPON resin. A series of ultrathin sections were prepared by using a Leica UCT ultramicrotome (Leica Microsystems), counterstained with uranyl acetate and lead citrate, and subsequently examined in a JEOL TEM 1010 operated at 80 kV. The TEM images were false colorized in Adobe Photoshop software.

### Magnetic Yeast GPs' Separation of Macrophages

At 60 min after injection of mGPs, the flies were washed in PBS and homogenized in 600 mL of PBS using a pestle. The homogenate was sieved through a nylon strainer (40 μm). This strainer was then additionally washed with 200 μL of PBS, which was subsequently added to the homogenate subsequently. The samples were centrifuged (3 min, 4 °C, 3500 rpm), and the supernatant was washed with ice-cold PBS after each centrifugation (3 times). Prior to mGPs separation, samples were transferred to FACS polystyrene tubes by using a disposable bacterial filter (50 μm, Sysmex).

The macrophages were separated from the sample using the QuadroMACS Separator (Miltenyi Biotec) according to the manufacturer′s protocol. In brief, the magnetic LS column (Miltenyi Biotec) was placed in the QuadroMACS Separator and rinsed before isolation with equilibrative buffer (PBS, 0.5% BSA, 2 mM EDTA, pH 7.2). Subsequently, the sample with the cell suspension was loaded into the LS column, and the flow through was discarded. To wash off the remaining cells, the LS column was washed 3 times with 1 mL of equilibrative buffer (Miltenyi Biotec). To obtain the phagocytosing cells, the LS column was removed from the QuadroMACS Separator and washed with 2 mL of rinsing buffer (PBS, 0.5% BSA, 2 mM EDTA, pH 7.2), and the flow though was collected into a nuclease free Eppendorf tube.

### Analysis of Macrophage Viability after Magnetic Separation

The macrophages obtained by mGPs' separation were allowed to attach to the imaging slide for 25 min. Subsequently, the macrophages were fixed with 4% paraformaldehyde (PFA) in PBS (Polysciences). After 20 min, the samples were stained with Alexa Fluor Plus 405 Phalloidin (Invitrogen) for 40 min. Aqua polymount (Polysciences) was used to mount the sample. The macrophages were imaged using an Olympus FluoView 3000 confocal microscope. Apart from visual assessment of cytoskeleton remodeling, cell viability was also determined quantitatively by letting the isolated macrophages spread on the surface of the Neubauer counting chamber, staining by trypan blue in a ratio of 1:1 to a final concentration of 0.02%, and counting.

### FACS Isolation of Macrophages

As a reference experiment, the GFP-expressing macrophages were isolated from CrqGal4 > GFP male flies using fluorescence-activated cell sorting (FACS). Three hundred flies were anesthetized with CO_2_, washed in PBS, and homogenized in 600 mL of PBS using a pestle. The homogenate was sieved through a nylon cell strainer (40 μm). This strainer was then additionally washed with 200 μL of PBS, which was added to the homogenate subsequently. The samples were centrifuged (3 min, 4 °C, 800*g*), and the supernatant was washed with ice-cold PBS after each centrifugation (3 times). Prior to sorting, samples were transferred to FACS polystyrene tubes using a disposable bacterial filter (50 μm, Sysmex), and macrophages were sorted into 100 μL of PBS using a S3TM Cell Sorter (BioRad). Isolated cells were verified by fluorescence microscopy and differential interference contrast.

### Gene Expression Analysis

Gene expression analysis was performed on 100 000 isolated macrophages. The macrophages were isolated by a cell sorter (S3e Cell Sorter, BioRad) as described in the section Isolation of Macrophages, transferred to TRIzol Reagent (Invitrogen), and homogenized using a DEPC-treated pestle. Subsequently, RNA was extracted with TRIzol Reagent (Invitrogen) according to the manufacturer’s protocol. Superscript III Reverse Transcriptase (Invitrogen) primed by an oligo(dT)20 primer was used for reverse transcription. Relative expression rates for particular genes were quantified on a CFX 1000 Touch Real-Time Cycler (BioRad) using the TP 2× SYBR Master Mix (Top-Bio) in three technical replicates with the following protocol: initial denaturation −3 min at 95 °C, amplification −15 s at 94 °C, 20 s at 56 °C, and 25 s at 72 °C for 40 cycles. Melting curve analysis was performed at 65–85 °C/step 0.5 °C. The qPCR data were analyzed using double delta Ct analysis, and the expressions or specific genes were normalized to the expression of Ribosomal protein 49 (Rp49) in the corresponding sample. The relative values (fold change) to the control are shown in the graphs. Samples for gene expression analysis were collected from three independent experiments.

### Primer Sequences

Rp49 forward: AAGCTGTCGCACAAATGGCG^30,31^
Rp49 reverse: GCACGTTGTGCACCAGGAAC
Hemolectin forward: GCGTACGAAGGAGATTCTC
Hemolectin reverse: CACCTCGTGCTTCTGTGT
Croquemort forward: CTTCTGGCCGGGTATTGCAG
Croquemort reverse: GCTTTCATAGGCATCAGT
Lactate dehydrogenase forward: CAGAGAAGTGGAACGAGCTG
Lactate dehydrogenase reverse: CATGTTCGCCCAAAACGGAG
Basket forward: TACGGCCCATAGGATCAGGT
Basket reverse: CCCTATATGCTCGCTTGGCA
Relish forward: ACAGGACCGCATATCG
Relish reverse: GTGGGGTATTTCCGGC
Diptericin A forward: GCTGCGCAATCGCTTCTACT
Diptericin A reverse: TGGTGGAGTGGGCTTCATG
Defensin forward: GTTCTTCGTTCTCGTGG
Defensin reverse: CTTTGAACCCCTTGGC
Metchnikowin forward: AACTTAATCTTGGAGCGA
Metchnikowin reverse: CGGTCTTGGTTGGTTAG
Drosocin forward: CCATCGTTTTCCTGCT
Drosocin reverse: CCATCGTTTTCCTGCT
Enolase forward: CAACATCCAGTCCAACAAGG
Enolase reverse: GTTCTTGAAGTCCAGATCGT
Phosphofructosekinase forward: AGCTCACATTTCCAAACATCG
Phosphofructosekinase reverse: TTTGATCACCAGAATCACTGC
Phosphoglucose isomerase forward: ACTGTCAATCTGTCTGTCCA
Phosphoglucose isomerase reverse: GATAACAGGAGCATTCTTCTCG
Unpaired3 forward: AGAACACCTGCAATCTGAAGC
Unpaired3 reverse: TCTTGGTGCTCACTGTGGCC
Imaginal morphogenesis protein late 2 forward: TTCGCGGTTTCTGGGCACCC
Imaginal morphogenesis protein late 2 reverse: GCGCGTCCGATCGTCGCATA
Eiger forward: AGCTGATCCCCCTGGTTTTG
Eiger reverse: GCCAGATCGTTAGTGCGAGA
Stat92E forward: CTGGGCATTCACAACAATCCAC
Stat92E reverse: GTATTGCGCGTAACGAACCG.

## Results and Discussion

### Physicochemical Properties of mGPs

**Figure 2:**
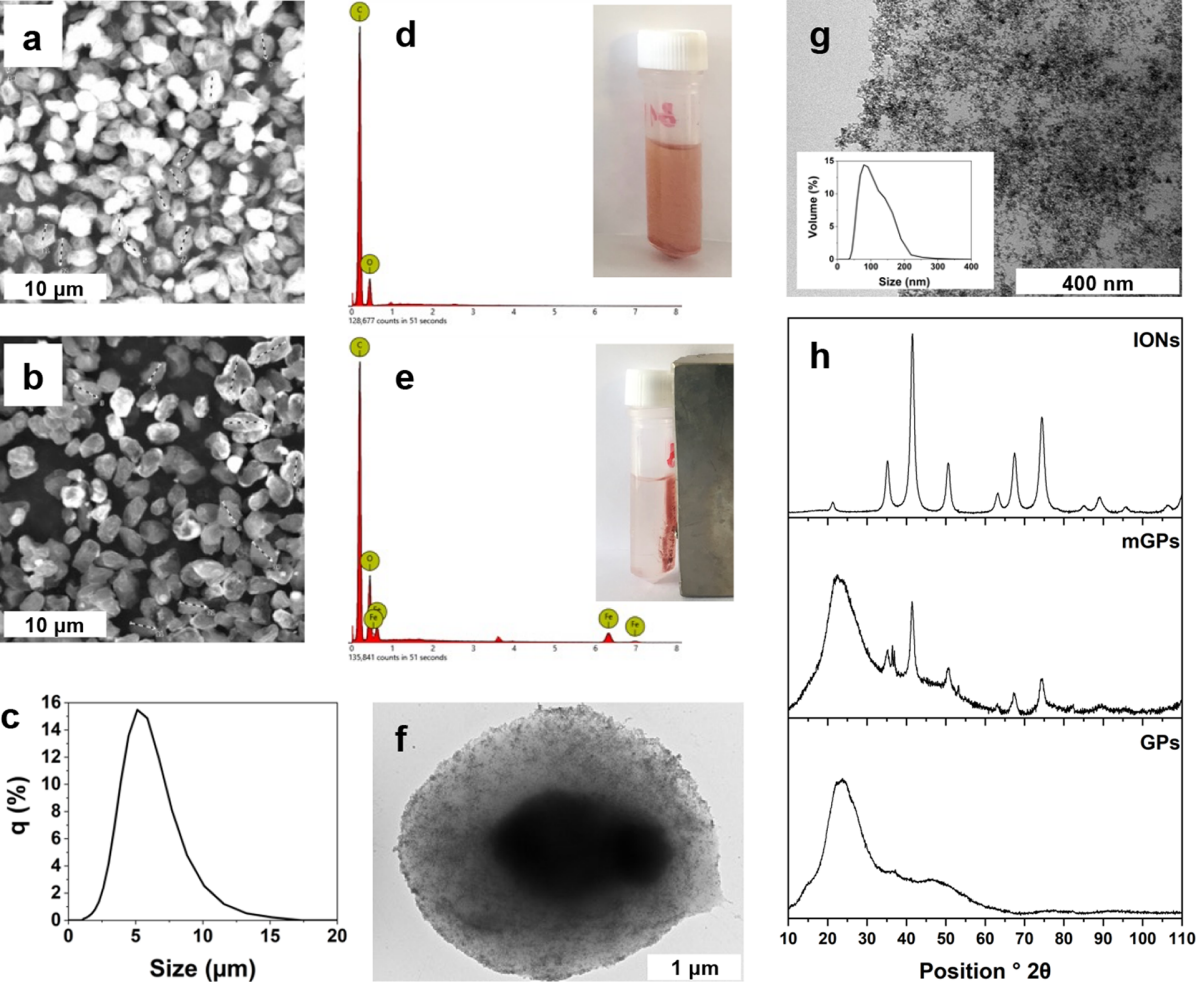
(a) SEM of plain glucan particles. (b) SEM of mGPs. The scale bars in both SEMs are 8 μm. (c) Particle size distribution of mGPs in water, measured by static light scattering; the volume-mean particle size is 5.1 ± 1.9 μm. (d) EDX spectrum of plain GPs. (e) EDX spectrum of mGPs, proving the presence of iron. The macroscopic manifestation of the presence of iron oxide in mGPs is their attraction to a magnet as shown in the inset. (f) TEM of a single mGP. The scale bar represents 1000 nm. (g) Detailed TEM showing how IONs are entrapped and uniformly dispersed within the mGP shell. The scale bar represents 200 nm. The volume-weighted particle size distribution of dextran-coated IONs in water before incorporation into mGPs, measured by DLS, is shown as inset. (h) XRPD spectra of IONs, plain GPs, and mGPS, proving the presence of iron oxide in mGPs.

After the incorporation of IONs by spray drying, mGPs retained the characteristic wrinkled ellipsoid shape known from plain GPs (Figure 2a,b). The volume-mean particle size of mGPs determined by laser diffraction was 5.1 ± 1.9 μm (Figure 2c), which is consistent both with the size of original yeast and with the values previously reported for unmodified GPs.^14^ The fact that the incorporation of magnetic particles did not cause aggregation or changes in the surface morphology of mGPs is crucial for subsequent uptake by phagocytosing cells. Energy-dispersive X-ray spectroscopy (EDX) of plain and mGPs (Figure 2d,e) proved the presence of IONs in mGPs. The Fe content of mGPs determined by EDX was 1.4% (Table 1). The iron content determined independently by AAS was 1.2 ± 0.1%. TEM analysis revealed that IONs were uniformly distributed within the polysaccharide shell of mGPs (Figure 2f). Prior to their incorporation into mGPs, dextran-coated IONs had a volume-mean diameter of 124.1 nm (measured by DLS in water) with a polydispersity index of 0.144 (Figure 2g inset). After incorporation into mGPs, IONs remained well dispersed within the glucan shell (Figure 2g). Note that the individual iron oxide cores visible as darker spots in the TEM image are smaller than the equivalent hydrodynamic diameter of fully hydrated dextran-coated IONs measured by DLS. This is because the dextran coating is not distinguishable from the beta-glucan background and also because magnetic nanoparticles are known to form temporary clusters in aqueous media.

**Table 1:** EDX Analysis of mGPs

sample	element symbol	atomic number	atomic concentration %
plain GPs	C	6	81.7
	O	8	18.3
	Fe	26	0.0
mGPs	C	6	76.9
	O	8	21.7
	Fe	26	1.4

The presence of iron oxide in the composite mGPs was additionally proven by measuring the XRPD spectra (Figure 2h). The characteristic crystalline peaks of iron oxide at 21.5°, 35.1°, 67.3°, and 74.4° 2θ were clearly visible in mGPs, while no such peaks were present in plain GPs. A crucial feature with respect to further application is the stability of mGPs in aqueous media in terms of ION retention. To detect potential loss of IONs during magnetic manipulation in an aqueous medium, mGPs were repeatedly separated by a magnet and redispersed. No free IONs could be detected in the supernatant, indicating that the embedding of IONs in the polysaccharide shell of mGPs was sufficiently strong to prevent the loss of magnetic properties over time. The full characterization of the magnetic properties of IONs including magnetization curves at 5 and 300 K and field-cooled and zero-field-cooled susceptibility have been reported in our recent work.^29^ The macroscopic manifestation of their magnetic properties is the ability to attract mGPs to a permanent magnet and separate them from solution, as shown in Figure 2.

### Biodistribution and Macrophage Uptake of mGPs

**Figure 3:**
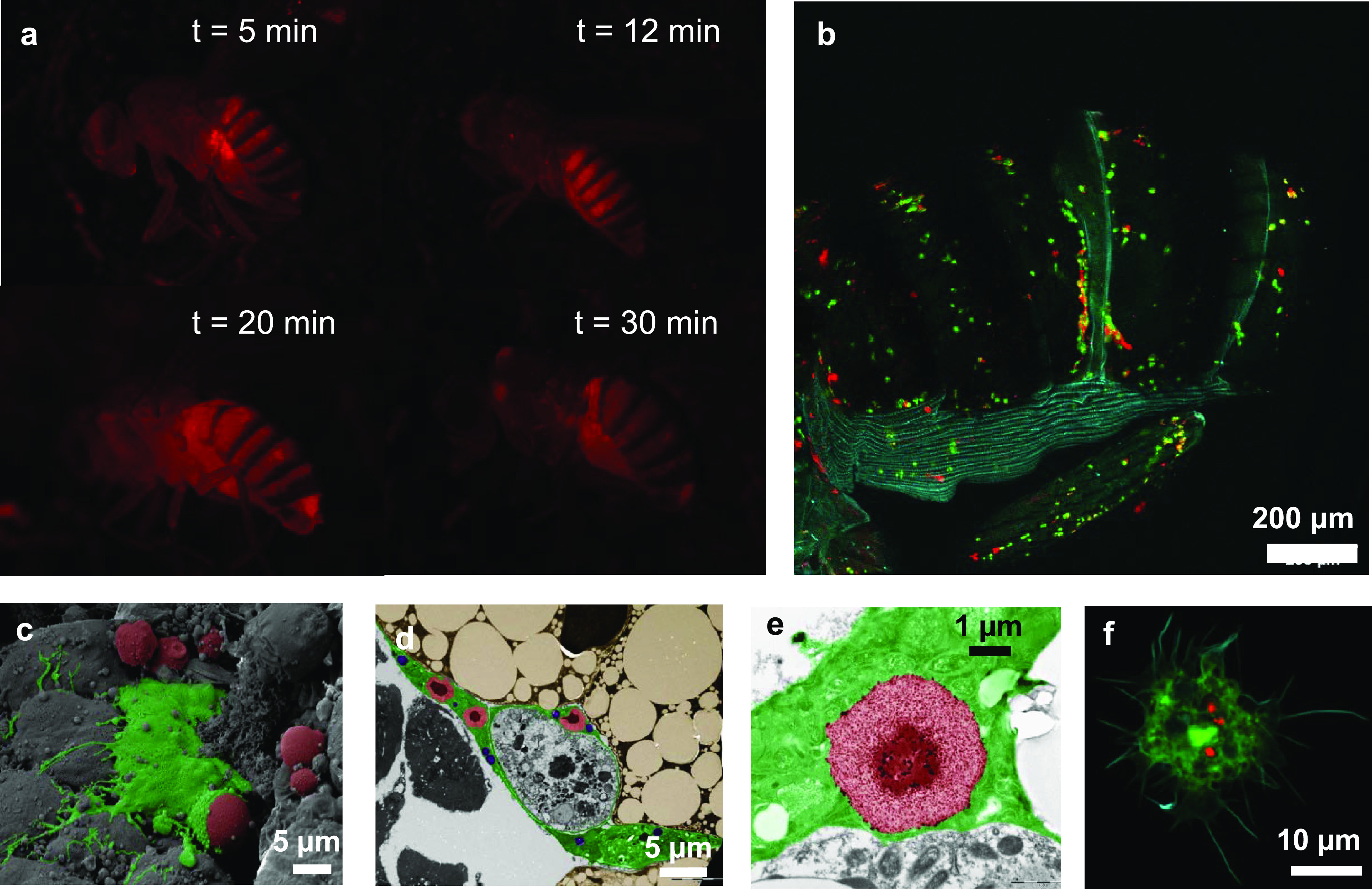
(a) Time progress of mGP biodistribution in *Drosophila* after injection. Within 20 min, mGPs reach even distal parts of the body of adult flies. (b) Distribution of mGPs (red) in adult *Drosophila* at 1 h after injection, showing colocalization with macrophages (green). (c) Pseudocolored SEM micrograph showing the process of engulfment of mGPs (red) by a macrophage (green) at 20 min after injection. (d) Pseudocolored TEM micrograph showing the localization of endocytosed mGPs (red) in the macrophages (green) at 1 h after injection. (e) TEM micrograph showing the detail of an endocytosed mGP (red) in the cytosol of the *Drosophila* macrophage (green). (f) Representative confocal image of a phagocytosing cell (green) from a CrqGal4 > GFP adult *Drosophila* injected by mGPs (red) at 1 h after injection. Actin was stained by phalloidin (cyan).

For investigating the biodistribution of mGPs and subsequent magnetic separation of viable macrophages, a *Drosophila melanogaster* strain bearing an endogenous construct for GFP protein expression in macrophages (Crq > Gal4; UAS2xGFP) was employed. Such macrophages are easily recognized for assaying their morphology and counting. The injection of 0.1% w/w mGPs led to a fast systemic distribution through the opened circulatory system of the fly (Figure 3a). Within 20–30 min after injection, mGPs could be found throughout the body of adult *Drosophila* including the distal parts. Within 1 h after injection, clear colocalization in areas occupied by macrophages was observable (Figure 3b), which is consistent with the in vivo behavior of plain GPs reported earlier. The internalization of mGPs by macrophages has been proven by the analysis of whole-body cross sections by SEM and TEM (Figure 3c–e). Analysis of dissected immune cells revealed that macrophages internalized multiple mGPs (Figure 3f). In a control experiment, free IONs (not encapsulated in mGPs) injected into adult flies were found not to specifically accumulate in macrophages (Figure S1, Supporting Information).

### Magnetic Cell Separation and Gene Expression

**Figure 4:**
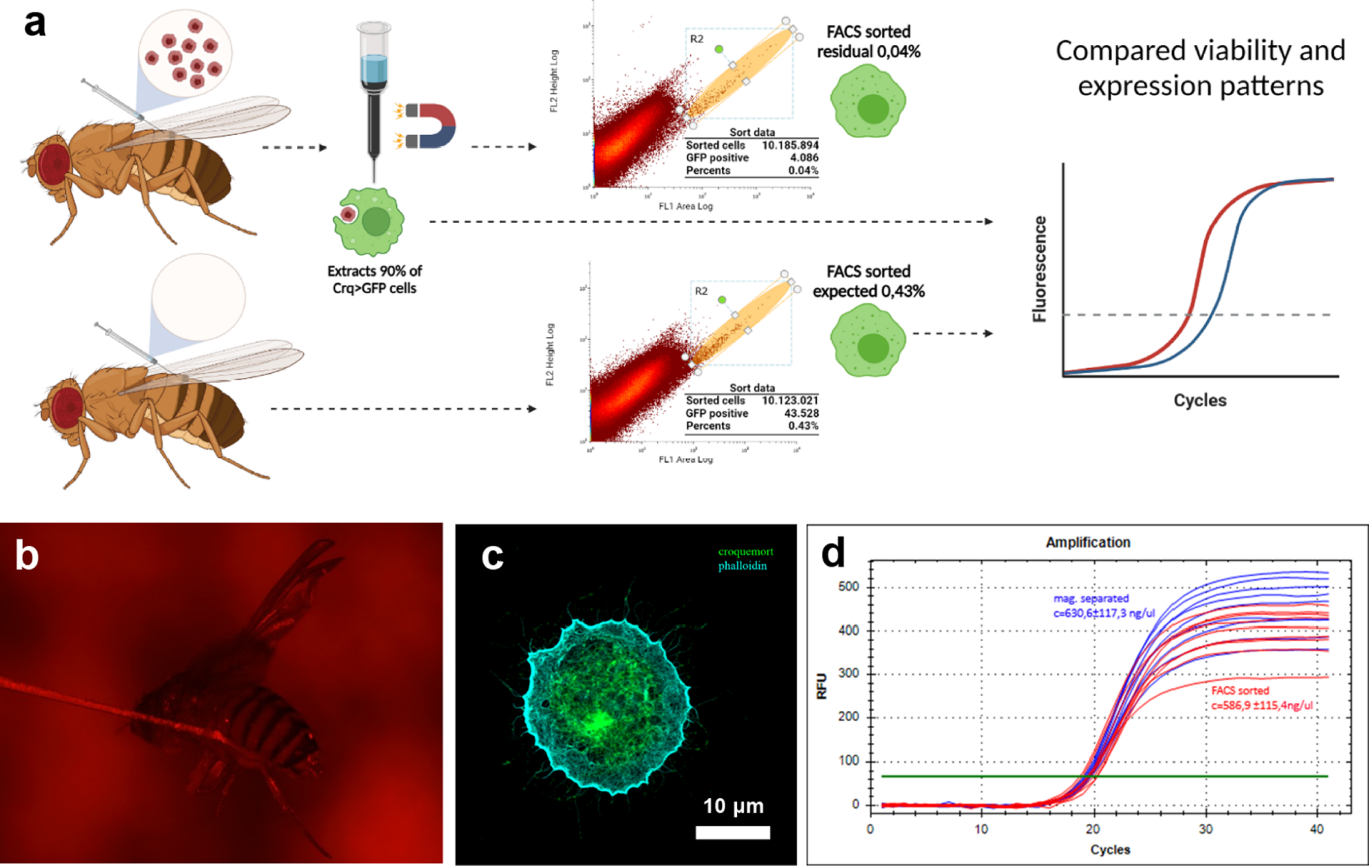
(a) Schematic representation of the cell separation process. Upper panel: CrqGal4 > GFP adult flies were injected with 50 nL of 0.1% (w/w) mGPs. The flies were homogenized, and the homogenate was magnetically sieved, resulting in the retention of approximately 90% of phagocytosing cells. The permeate was collected and FACS sorted based on the endogenously expressed GFP signal (G2 gate). The sorter detected the residual 10% of unseparated macrophages, constituting 0.04% out of the overall cell count. Lower panel: In a reference macrophage isolation experiment without mGP injection, the macrophages were sorted from the homogenate only by FACS, giving a yield of 0.46% out of the overall cell count (Table 2). The phagocytosing cells obtained by mGPs-based magnetic separation and FACS sorter show comparable viability and were subsequently used for RT-qPCR. (b) Visualization of the injection of adult fly with mGPs. (c) Confocal microscopy visualization of croquemort and phalloidin present in living macrophages after magnetic separation. (d) Quantification of the expression level of Rp49 (commonly used as a housekeeping gene in *Drosophila*) for magnetically and FACS-sorted macrophages.

Flies injected with mGPs were homogenized 45 min after particle administration, and the homogenates were used for magnetic column separation (QuadroMACS Separator, LS Columns, Miltenyi Biotec). In parallel, tissue homogenates from flies injected only with a buffer were processed by FACS separation of GFP-expressing macrophages as a control (Figure 4a). The statistical data accompanying Figure 4a based on four independent biological replicas are summarized in Table 2. Before magnetic separation, the homogenate contained 0.458% ± 0.049% of GFP-positive cells (macrophages). The residue after magnetic separation contained 0.042% ± 0.006% of GFP-positive cells, which represents approximately 9.3% of the original. Thus, magnetic separation was able to extract approximately 90.7% of all GFP-positive cells originally present in the homogenate, which is comparable to the yield obtained from FACS. The sensitivity of the method, defined as the fraction of macrophages targeted by mGP administration, was 97.9% ± 2.5% (*N* = 90; 4 replicates), while its selectivity, defined as the fraction macrophages within the population of cells that have engulfed mGPs, was 100% ± 0% (*N* = 100; 5 replicates). Details of the sensitivity and selectivity measurements are provided in Supporting Information. The subsequent isolation of RNA from samples obtained by both approaches provided a comparable amount of RNA (Figure 4d). This was confirmed by quantifying purified RNA on a nanodrop instrument and quantifying the expression level of Rp49, commonly used as a housekeeping gene in *Drosophila*. The concentration of Rp49 in the case of macrophages separated by mGPs and by FACS was 630.6 ± 117.3 and 586.9 ± 115.4 ng/μL, respectively.

**Table 2:** Sort Data Accompanying Figure 4a

before mag. separation	Rep.1	Rep.2	Rep.3	Rep.4	average	st. dev.
sorted cells	10,123,021	10,185,447	10,066,524	10,121,254	10,124,062	48,606
GFP positive	43,528	52,561	48,211	41,231	46,383	5040
percent	0.430	0.516	0.479	0.407	0.458	0.049

after mag. separation	Rep.1	Rep.2	Rep.3	Rep.4	average	st. dev.
sorted cells	10,185,894	10,024,653	10,144,874	10,132,241	10,121,916	68,768
GFP positive	4086	5112	3844	4117	4290	562
percent	0.040	0.051	0.038	0.041	0.042	0.006

**Figure 5:**
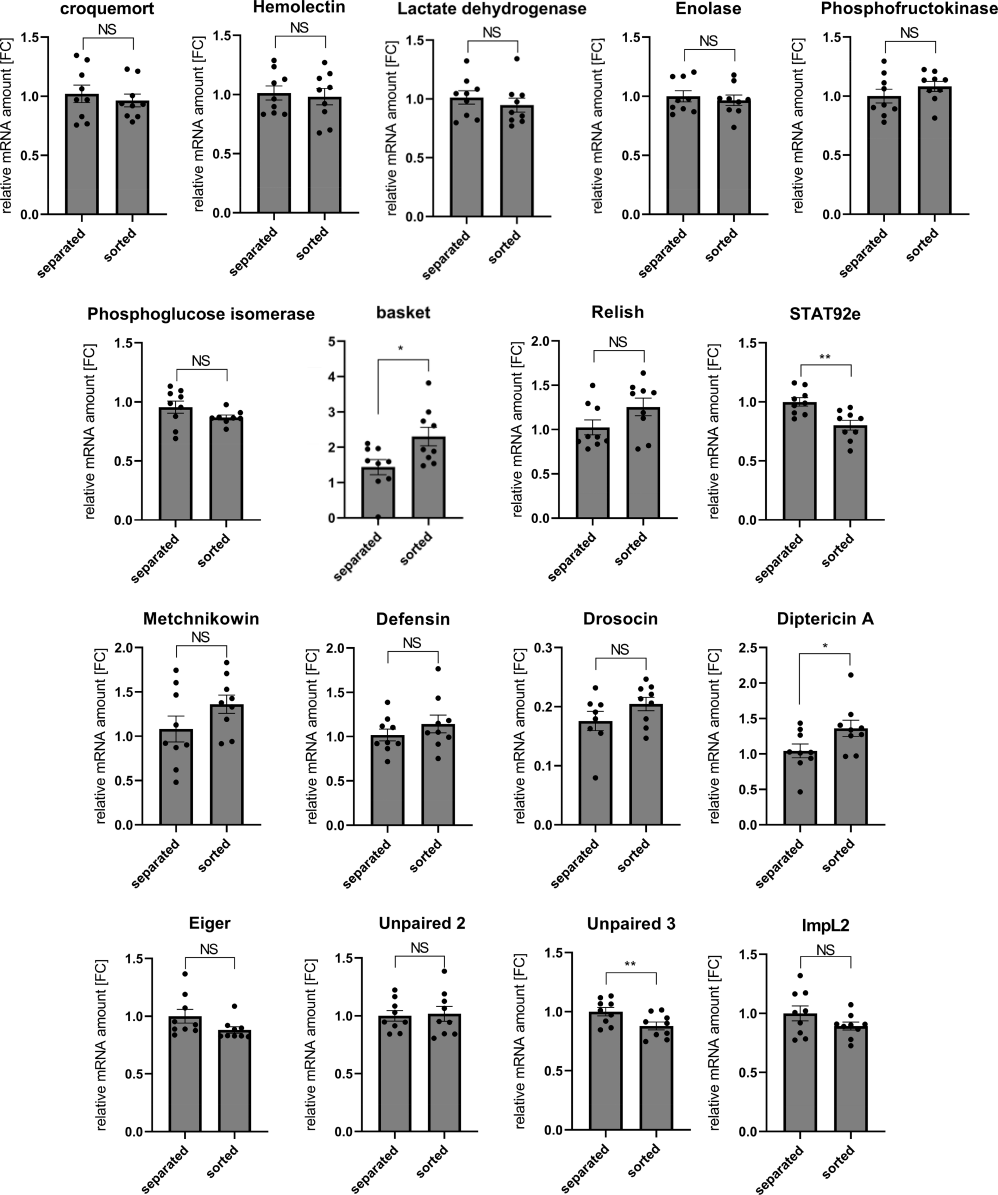
Comparison of gene expression of macrophage markers (croquemort, hemolectin), glycolytic gene (lactate dehydrogenase, enolase, phosphofructokinase, phosphoglucose isomerase), stress and immune response genes (basket, Relish, STAT92e), antimicrobial peptides (metchnikowin, diptericin A, drosocin, defensin), and cytokines (Eiger, Upd2, Upd3, ImpL2) in phagocytosing cells obtained by mGPs-based magnetic separation and FACS sorter. The results were compared by two-way ANOVA followed by Tukey’s multiple comparison test. Expression levels normalized against Rp49 are reported as fold change relative to the levels of the analyzed gene expression in mGPs-separated phagocytes, which were arbitrarily set to 1. The individual dots represent biological replicates with line/bar showing mean ± SD, asterisks mark statistically significant differences (*p < 0.05; **p < 0.01), and NS marks statistically insignificant differences.

The viability of the magnetically separated macrophages determined by the tryptophan blue assay was 95.5%. The good condition of the isolated cells manifested itself also by their characteristic spreading phenotype on the surface of a microscopic slide and cytoskeleton remodeling (Figure 4c). Finally, the expression level of macrophage-specific markers (hemolectin, croquemort), immune-related genes (defensin, drosocin, metchnikowin, diptericin A), and characteristic readout of cellular stress pathways (Relish, basket) were analyzed for both techniques, revealing that macrophages separated by means of magnetic glucan particles possess natural physiological features (Figure 5). This indicates that the mGP were not cytotoxic and their uptake did not cause any anomalous physiological response in the macrophages. The expression level of inflammatory cytokines was not found to be significantly different between magnetically separated and FACS-sorted macrophages (Figure 5), indicating that neither the engulfment of mGPs nor the magnetic separation process itself resulted in the activation of the inflammatory response. The macrophages separated by means of mGPs can in principle be subsequently used for various analyses such as gene expression analysis, metabolomics, proteomics, single-cell transcriptomics, and enzymatic activity analysis.^7,23−25^

## Conclusions

We have prepared mGPs using a new approach based on the deswelling of porous polysaccharide shell during rapid solvent evaporation during spray drying. This enables the irreversible entrapment of a large quantity of independently prepared IONs into the mGP structure, in which they remain homogeneously dispersed without undesired agglomeration of clustering. When injected into living *Drosophila*, mGP quickly spread across the body and were readily and selectively taken up by macrophages. This enabled subsequent macrophage isolation from tissue homogenates by a magnetic separation column.^26−28^ The key to the successful application of mGPs for magnetic cell separation were three properties: (i) preservation of the size, surface morphology and structural motifs characteristic of original GPs, which are a prerequisite for immune recognition and efficient phaogcytosis; (ii) high concentration of embedded IONs, which is a prerequisite for generating a sufficiently strong response of the particles to an external magnetic field; and (iii) biocompatibility, which is prerequisite for good viability and further application of the isolated cells without compromising normal cellular functions and gene expression.

Unlike magnetic separation based on attaching magnetic beads to the external cell surface via specific antibodies, the method based on mGPs has several advantages: (i) it enables antibody- and label-free isolation of immune cells; (ii) it covers all cells in the host organism that may participate in the engulfment of pathogens, with no need for knowing these cells a priori; (iii) due to a highly evolutionarily conserved feature (phagocytosis), the method can be used basically in all animals, not just insects; and (iv) the method allows short processing time, it is gentle, and the cells are exposed only to physiological buffers and no additional chemicals. Overall, it can be concluded that the fabrication of magnetic yeast GPs (mGPs) represents a suitable strategy for isolating macrophages, sufficient in amount and quality to perform gene expression analyses. Since this approach is independent of having endogenously expressed fluorescent markers or binding of cells via specific antibodies against the surface antigenic epitope, it may also be adapted for other situations where it is desirable to separate a population of live phagocytic cells from insect and noninsect species. Of course, it should also be noted that the presence of mGPs in the macrophages may not be universally desirable (e.g., when studying iron metabolism), but based on the data presented in this work (viability, functionality, and gene expression), the magnetically separated macrophages were not negatively affected by the engulfment of mGPs.

## Supporting Information Available

Results of a reference experiment in which free IONs (i.e., not embedded within mGPs) were injected into *Drosophila*; information on the evaluation of selectivity, sensitivity, and purity of the magnetic separation method; and information about the gating strategy used for cell sorting (PDF)
