# Supplementary material for: Magnetic Yeast Glucan Particles for Antibody-Free Separation of Viable Macrophages from Drosophila melanogaster
Source: ACS Biomater Sci Eng. 2023 Dec 4;10(1):355–64. doi: 10.1021/acsbiomaterials.3c01199 (PMC10777351; doi:10.1021/acsbiomaterials.3c01199)
Supplement: Supplementary file 1 — ab3c01199_si_001.pdf [file ab3c01199_si_001.pdf]

Supporting Information

Magnetic yeast glucan particles for antibody-free separation of viable macrophages from Drosophila melanogaster

Gabriela Krejčová [a#], Ivan Saloň [b#], Vojtěch Klimša [b], Pavel Ulbrich [c], Ayse Beyza Aysan [b], Adam Bajgar [ab*], František Štěpánek [b*]

[a] Department of Molecular Biology and Genetics, Faculty of Sciences, University of South Bohemia, Branišovská 1160/31, 37005 České Budějovice, Czech Republic

[b] Department of Chemical Engineering, University of Chemistry and Technology Prague, Technická 5, 166 28 Prague 6, Czech Republic

[c] Department of Biochemistry and Microbiology, University of Chemistry and Technology, Prague, Technická 5, 166 28 Prague 6, Czech Republic

[#] These authors contributed equally

[*] Corresponding authors: bajgaa00@prf.jcu.cz (A. Bajgar), stepanef@vscht.cz (F. Štěpánek)

SI-1: In vivo localisation of free IONs

After injecting adult *Drosophila* with the dextran coated iron oxide nanoparticles (IONs) of approximate 10 nm size their distribution was analysed throughout the body. Most of these particles were found in the colocalisation with *Drosophila* macrophages, however there was a significant accumulation of clustering magnetic particles in circulation, aortic region, and oenocytes (Fig. S1). Moreover, despite the substantial accumulation of these particles in macrophages, they did not produce enough magnetic force necessary for the magnetic separation of the cells.

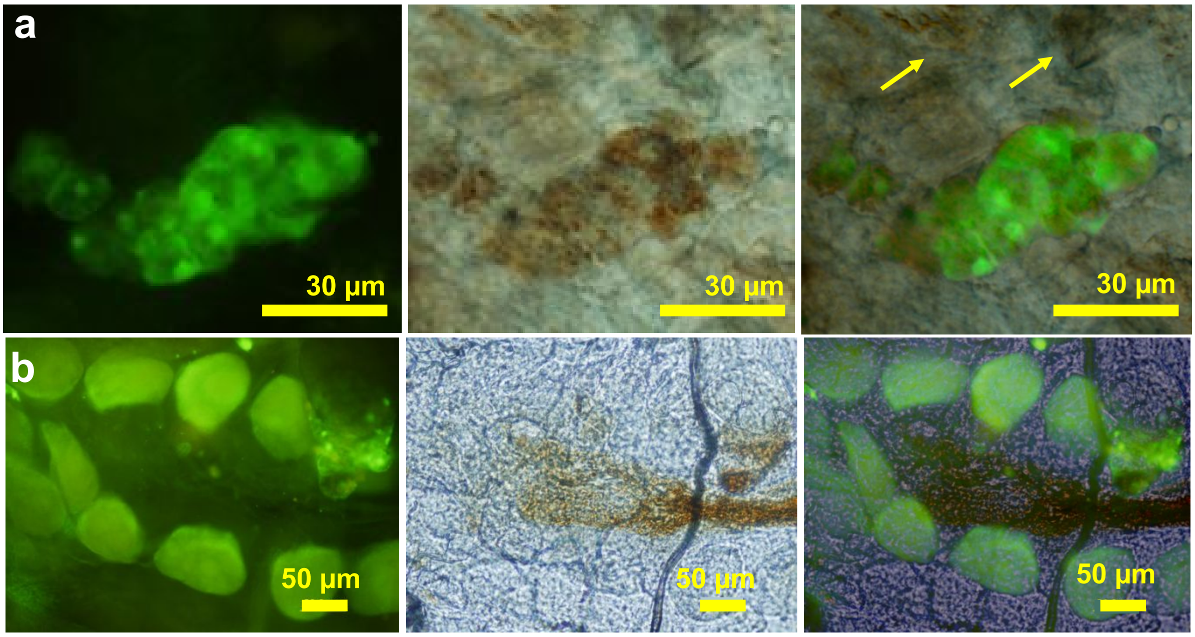

Fig. S1: a Injection of CrqGal4>GFP adult *Drosophila* by unbound Dex-SPIONs (brown) results in their ectopic localisation. Arrows show the localization of the nanoparticles outside the macrophages (green). **b** Ectopic deposition of magnetic nanoparticles (brown) in the aorta. Autofluorescence in the green channel was used to visualize the pericardial cells.

SI-2: Evaluation of Selectivity, Sensitivity and Purity

Sensitivity – what proportion of macrophages was targeted by mGP administration?

Experimental approach: flies bearing GFP positive macrophages (GREEN) were injected by Rhodamine-labeled GPs (RED). Cell-sorted macrophages based on GFP signal were subsequently inspected for the presence of Rhodamine-GPs by confocal microscopy. The experiment was done in four replicates, the percentage of cells containing GPs was as follows.

Sensitivity	GFP positive cells	containing RHOD.-GPs	Percent	Stdev.
	20	20	100.0	
	23	23	100.0	
	22	21	95.5	
	25	24	96.0	
Sum/sum/average/stdev.	**90**	**88**	**97.9**	**2.5**

Selectivity – to what extent could mGPs be engulfed by other cells than macrophages?

Experimental approach based on image analysis: TEM images of the mGP-injected flies were inspected on the distribution of mGPs throughout the body. Result: from 100 inspected TEM images, mGPs were found only in macrophages-like cells. mGPs were found in 142 macrophage-like cells in all inspected TEM images (N=150), that means in 94.7%.

Experimental approach based on fluorescence: flies carrying GFP-positive macrophages (GREEN) were injected by both mGPs (no color) and phagocytic marker Sa-pHrodo (RED). Cells containing mGPs were isolated and inspected for the presence of phagocytic marker by confocal microscopy. Nuclei of all isolated cells were labeled by DAPI (BLUE).

Selectivity	mGPs-separated cells	pHrodo content	Percent	Stdev.
	25	25	100	
	25	25	100	
	25	25	100	
	25	25	100	
Sum/sum/average/stdev.	**100**	**100**	**100**	**0**

Purity – how many of mGP separated cells are GFP positive and how many of them are macrophages?

Experimental approach: Flies were co-injected with mGPs and independent phagocytic marker (S.a. pHrodo, giving the red fluorescent signal). After magnetic cell separation, the fraction of cells displaying the signal of phagocytic marker was calculated. Result: 100% of mGP separated cells display phagocytosis of S.a. pHrodo marker, from which 98.0%±2.3% were GFP positive (N=100; 4 replicates).

Purity	mGPs-separated cells	GFP signal	Percent	Stdev.
	25	24	96	
	25	25	100	
	25	24	96	
	25	25	100	
Sum/sum/average/stdev.	**100**	**98**	**98.0**	**2.3**

SI-3: Gating strategy

The gating strategy was based on the comparison of fly strain bearing GFP-positive macrophages and controls. In the first window shown below, the duplets and bigger cell clusters were excluded in side- and forward-scatter scanners. GFP-positive macrophages were detected as a cloud of cells missing in the negative controls (genetically related flies missing the GFP-reporter). The identity of cells was confirmed by microscopy observation of GFP signal and the ability of these cells to spread on microscopy glass and phagocyte S.a. pHrodo labelled particles. Negative is shown on the left, positive on the right in Fig. S2 bellow.

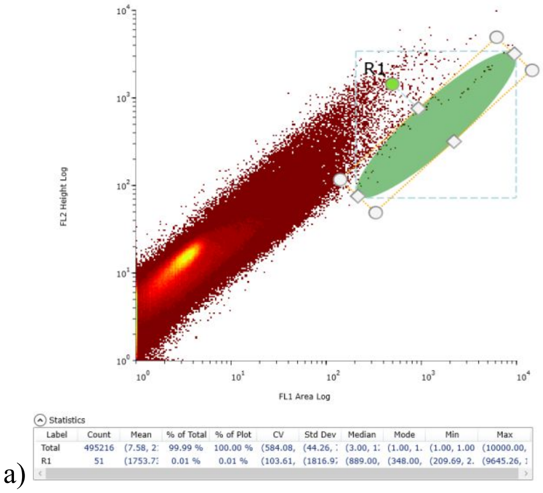

a)

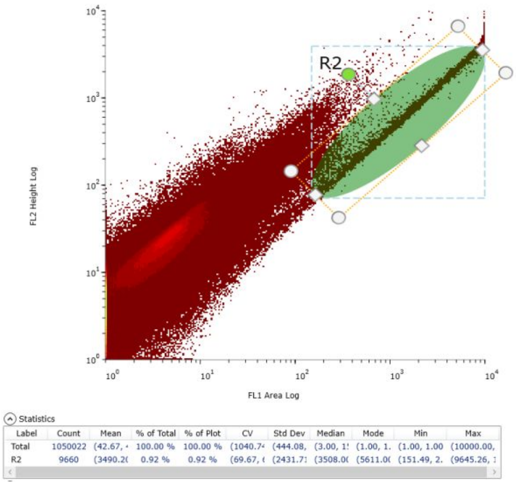

b)

Fig. S2: Sort gating for GFP-expressing macrophages. a) negative, b) positive.
